# Gastritis and gastroesophageal reflux disease are strongly associated with non-allergic nasal disorders

**DOI:** 10.1186/s12890-020-01364-8

**Published:** 2021-02-08

**Authors:** Eliana Finocchio, Francesca Locatelli, Francesca Sanna, Roberta Vesentini, Pierpaolo Marchetti, Gianluca Spiteri, Leonardo Antonicelli, Salvatore Battaglia, Roberto Bono, Angelo Guido Corsico, Marcello Ferrari, Nicola Murgia, Pietro Pirina, Mario Olivieri, Giuseppe Verlato

**Affiliations:** 1 Section of Epidemiology and Medical Statistics, Department of Diagnostic and Public Health, University of Verona, Strada Le Grazie, 8, 37134 Verona, Italy; 2 Unit of Occupational Medicine, Department of Diagnostics and Public Health, University of Verona, P.le L.A. Scuro 10, 37134 Verona, Italy; 3 Department of Internal Medicine Ospedali Riuniti Ancona, Via Conca, 71, 60126 Ancona, Italy; 4 University of Palermo, Piazza Marina, 61, 90133 Palermo, Italy; 5 Department of Public Health and Pediatrics, University of Torino, Via Santena 5 bis, 10126 Torino, Italy; 6 Department of Internal Medicine and Medical Therapy, University of Pavia, Palazzo Botta, 10, 27100 Pavia, Italy; 7 Unit of Respiratory Diseases, Department of Medicine, University of Verona, P.le L.A. Scuro 10, 37134 Verona, Italy; 8 Section of Occupational Medicine, Respiratory Diseases and Toxicology, University of Perugia, Piazza dell’Università, 1, 06123 Perugia, Italy; 9 Department of Clinical, Surgical and Experimental Sciences, University of Sassari, Piazza Università, 21, 07100 Sassari, Italy

**Keywords:** Gastritits, Gastroesophageal reflux disease, Allergic rhinitis, Non-allergic rhinitis, Sinusitis

## Abstract

**Background:**

Gastroesophageal reflux disease (GERD) has been reported to be significantly associated with chronic rhinosinusitis, but the strength of the association is still debated.

**Aims:**

To evaluate the strength of the association between gastritis/GERD and non-allergic rhinitis (NAR)/allergic rhinitis (AR)/sinusitis.

**Methods:**

We investigated 2887 subjects aged 20–84 years, who underwent a clinical visit in seven Italian centres (Ancona, Palermo, Pavia, Terni, Sassari, Torino, Verona) within the study on Gene Environment Interactions in Respiratory Diseases, a population-based multicase-control study between 2008 and 2014. Subjects were asked if they had doctor-diagnosed “gastritis or stomach ulcer (confirmed by gastroscopy)” or “gastroesophageal reflux disease, hiatal hernia or esophagitis”. The association between NAR/AR/sinusitis and either gastritis or GERD was evaluated through relative risk ratios (RRR) by multinomial logistic regression.

**Results:**

The prevalence of gastritis/GERD increased from subjects without nasal disturbances (22.8% = 323/1414) to subjects with AR (25.8% = 152/590) and further to subjects with NAR (36.7% = 69/188) or sinusitis (39.9% = 276/691). When adjusting for centre, sex, age, education level, BMI, smoking habits and alcohol intake, the combination of gastritis and GERD was associated with a four-fold increase in the risk of NAR (RRR = 3.80, 95% CI 2.56–5.62) and sinusitis (RRR = 3.70, 2.62–5.23) with respect to controls, and with a much smaller increase in the risk of AR (RRR = 1.79, 1.37–2.35)..

**Conclusion:**

The study confirmed the association between gastritis/GERD and nasal disturbances, which is stronger for NAR and sinusitis than for AR.

## Background

Rhinitis is a global common problem and is defined as the presence of at least one of the following: congestion, rhinorrhea, sneezing, nasal itching, and nasal obstruction. The two major classifications are allergic (AR) and non-allergic rhinitis (NAR) [1]. NAR occurs when obstruction and rhinorrhea are related to non-allergic, non-infectious triggers such as a change in the weather, exposure to caustic odors or cigarette smoke, barometric pressure differences, etc. [2]. The prevalence of AR in adults in Europe ranged from 17 to 28.5% [3], while NAR affects up to 30% of individuals in the Western population [4].

Gastro-esophageal reflux disease (GERD) is also a worldwide prevalent condition, which is on the rise in Europe and North America [5]. Hence also esophageal and extraesophageal diseases associated with GERD are expected to increase. Some of the well-established extraesophageal manifestations are reflux-induced cough, laryngitis, asthma and dental erosion. Other manifestations, such as sinusitis, pharyngitis, idiopathic pulmonary fibrosis, and recurrent otitis media, are proposed but not established as it is unclear whether GERD is a significant causal or exacerbating factor [6].

Since both chronic rhinosinusitis and GERD are highly prevalent, it is difficult to establish a direct relation between them, as they can easily coexist independently [7]. Moreover, while the association between GERD and nasal disorders gained support in children [8, 9], in adults the evidence is still sparse. Several studies focused on the possible correlation of GERD and sinusitis [10–12], which have been reported to occur together more frequently than expected [13]. At first, several reviews did not find out a clear evidence-based relationship between Chronic rhinosinusitis and GERD [13–15]. However, in the last years the association between GERD and sinusitis gained support. Two European studies found that the SNOT (Sino-Nasal Outcome Test) score significantly increased among patients with GERD, suggesting a direct role of GERD in the development of chronic rhinosinusitis [10, 16]. A large cohort study based on Taiwan Health Care Utilization database by Lin et al. [17] found that the risk of developing chronic rhinosinusitis was more than doubled in cases with newly diagnosed GERD with respect to controls matched for sex, age and comorbidities. In a population-based Brazilian survey the diagnosis of gastritis/ulcer/gastroesophageal reflux was associated with higher prevalence of rhinosinusitis symptoms in multivariable analysis [18]. An Italian study on a small series undergoing both nasal cytology and esophageal manometry and 24-h pH-impedance monitoring showed that NAR with neutrophils strongly correlated with higher acid exposure time and refluxes number [19]. On the basis of this accumulating evidence, the International Consensus Statement on Allergy and Rhinology: Rhinosinusitis [20] assigned grade B evidence to support the association between chronic rhinosinusitis and GERD, although causation could not be clearly demonstrated.

The association between GERD and AR is more questioned. The recent International Consensus Statement on Allergic Rhinitis does not even mention GERD at all as a potential risk factor [21]. The situation is complex, as several studies which found an association between GERD and nasal disorders did not distinguish between NAR and AR [22].

Since nasal disorders are highly prevalent diseases that can have a deep impact on individual life and healthcare system, it is important to identify causative and triggering factors, and their comorbidities. The present study aimed to investigate the relation between gastritis/GERD and allergic and non-allergic rhinitis in a large population-based case–control study.

## Methods

### Study design

The study was performed in the frame of the GEIRD (Gene-Environment Interactions in Respiratory Diseases) study, a multicase-control study on respiratory health, involving seven Italian centers, three located in Northern Italy (Verona, Pavia, Turin), two in Central Italy (Ancona, Terni) and two in the major islands (Sassari in Sardinia and Palermo in Sicily) [23]. The study comprised a screening phase and a clinical phase. In the screening phase a screening questionnaire was mailed to random samples from the general population aged 20–84 years, while in the clinical phase subjects reporting symptoms suggestive of chronic bronchitis, asthma or rhinitis, as well as a sample of subjects without respiratory symptoms, were invited to a local Respiratory/Allergy Unit, in order to undergo interviews and clinical tests. In particular, participating subjects were administered a modified version of the ECRHS (European Community Respiratory Health Survey) clinical questionnaire, including detailed questions on socio-demographic characteristics, smoking habits and other lifestyle factors, respiratory symptoms and other disturbances, drug consumption [23; available at www.geird.org]. In each center, the GEIRD study was approved by the local ethics committee and written consent was obtained from each participant.

Fifty-nine percent (17,972/30,349) of selected subjects answered the screening questionnaire, while 2,945 subjects out of 7,739 participated in the clinical visit between 2008 and 2014, yielding a participation rate of 40.1%.

### Nasal disorders

Subjects were classified as having "rhinitis" if they answered affirmatively to at least one of the following questions: "Do you have any nasal allergies including hay fever?", "During your lifetime have you ever had any nasal allergies including hay fever?", "Have you ever had a problem with sneezing, or a runny or a blocked nose when you did not have a cold or the flu?". Rhinitis was further classified as allergic rhinitis (AR) and non-allergic rhinitis (NAR), according to the presence or absence of atopy. Subjects were also asked whether they ever suffered from nasal polyps.

Treatments for nasal disorders were assessed by the following questions "Have you used any of the following nasal medicines (e.g. nasal sprays, inhaled powders or drops) for the treatment of your nasal disorders?" and "Have you used any of the following pills, capsules, or tablets for the treatment of your nasal disorder?".

### Other respiratory disorders

Asthma was deemed present when the subject reported physician-diagnosed asthma. The disease was further classified in: current asthma if the subject took any medicine for asthma or had had an attack of asthma or reported any asthma-like symptom (wheezing, chest tightness or shortness of breath) in the previous 12 months; past asthma otherwise.

Chronic cough and phlegm was defined by a positive answer to the question: “*Have you had coughing and phlegm on most days for a minimum of 3 months a year and for at least 2 successive years*?”. Doctor-diagnosed chronic bronchitis was defined by an adfirmative answer to the question: “*Have you ever been told by a doctor that you have or had chronic bronchitis, chronic obstructive pulmonary disease (COPD) or emphysema*?”.

### Atopy

Atopy was established by a positive skin prick test in which the following panel of allergens were used: Cupressus arizonica, Dermatophagoides pteronyssinus, Artemisia vulgaris, Dermatophagoides farinae, Ambrosia artemisifolia, Alternaria tenuis, Parietaria judaica, dog dandruff, Corylus avellana, cat, Olea europea, Betula verrucosa, Cladosporium herbarum, and Phleum pratense. The result was considered positive if, after twenty minutes, the average wheal diameter was 3 mm greater than the negative control.

### Gastritis/gastroesophageal reflux

Subjects were classified as having gastritis if they answered positively to the question "*Has a doctor told you having or have had gastritis or stomach ulcer (confirmed by a gastroscopy)*?" Similarly, subjects were considered having gastroesophageal reflux disease (GERD) if they answered affirmatively to the question "*Has a doctor told you having or have had gastroesophageal reflux disease, hiatal hernia or esophagitis?*”.

### Lifestyle factors

Subjects were classified as normal weight (BMI < 25 kg/m^2^), overweight (25 ≤ BMI < 30 kg/m^2^), or obese (BMI ≥ 30 kg/m^2^). They were considered active when reporting to exercise for at least 1 h getting out of breath or sweating with a frequency of at least 2–3 times a week.

With regard to smoking habits, subjects were classified as (1) current smokers, if they reported to have smoked at least one cigarette per day or one cigar a week for as long as one year, and also in the last month; (2) ex-smokers if they had smoked the same minimum amount previously reported, but had stopped smoking for at least one month before the interview; (3) never smokers otherwise. As regards alcohol consumptions, subjects were classified as drinkers and nondrinkers.

### Statistical analyses

Significance of the association between AR/NAR/sinusitis and potential risk factors was evaluated by Fisher's exact test or Chi-squared test. The same statistical tests were used to evaluate the association between gastritis/gastroesophageal reflux and other risk factors.

Multivariable analysis was accomplished by a multinomial logistic regression model [24], where the response variable was nasal symptoms: 0 = no symptom (base outcome), 1 = allergic rhinitis, 2 = non-allergic rhinitis, 3 = sinusitis. Gastroesophageal reflux (none/gastritis/reflux/both gastritis and reflux) was the explanatory variable, while sex, age (per 10 year increase), age at completing full-time education (< 18, 18–21, ≥ 22 years), BMI (< 25, 25–29.9, ≥ 30 kg/m^2^), smoking habits (never smoker, past smoker, current smoker), alcohol intake (nondrinker, drinker), were the potential confounders. Results were synthesized through the relative risk ratios (RRR), adjusting standard errors for intra center correlation. Analyses were performed with STATA statistical software, release 14 (StataCorp, College Station, TX, USA) and statistical significance was set at p < 0.05.

## Results

### Description of controls and cases of AR/NAR/sinusitis, as a function of main risk factors

2887 subjects participated in the clinical visit and they had a mean age (SD) of 50.1 (13.2) years. Cases of AR and sinusitis were younger (mean age ± SD = 46.0 ± 11.8 and 48.7 ± 11.9 years, respectively) than controls and cases of NAR (52 ± 13.7 and 54.6 ± 14 years, respectively) (p < 0.001). Controls and cases of NAR had a lower level of education and physical activity, a higher prevalence of obesity than cases of AR and sinusitis. Atopy, which was used to define NAR and AR, had a prevalence of 27% in controls and 58% in cases of sinusitis. Nasal polyps were rare in controls and cases of NAR, and more common in cases of AR and sinusitis. Use of antihistamines and steroids was frequent among cases of AR, and rare among cases of NAR, while the use of vasoconstrictors was similar in the two groups. Sex, smoking habits and alcohol intake did not significantly differ between cases and controls (Table 1).

**Table 1:** Number and percent of controls, cases of non-allergic rhinitis (NAR), allergic rhinitis (AR) and sinusitis as a function of main socio-demographic, lifestyle, and clinical characteristics

	Controls (n = 1416) n(%)	AR (n = 592) n(%)	NAR (n = 188) n(%)	Sinusitis (n = 691) n(%)	*p *value
Sex					0.068
Male	729 (51.5)	317 (53.5)	87 (46.3)	326 (47.2)	
Female	687 (48.5)	275 (46.5)	101 (53.7)	365 (52.8)	
Age (years)					< *0.001*
20–34	144 (10.2)	*95 (16.1)*	12 (6.4)	70 (10.1)	
35–44	339 (23.9)	*194 (32.8)*	34 (18.1)	*218 (31.6)*	
45–54	388 (27.4)	*191 (32.3)*	*61 (32.5)*	*218 (31.6)*	
55–64	254 (17.9)	72 (12.2)	*36 (19.2)*	119 (17.2)	
≥ 65	*291 (20.6)*	40 (6.8)	*45 (23.9)*	66 (9.6)	
Time at stopping education (years)					< *0.001*
< 18	*465 (33.1)*	141 (24)	*64 (34.8)*	190 (27.7)	
18–21	498 (35.5)	*222 (37.8)*	*68 (37)*	*267 (38.9)*	
≥ 22	440 (31.4)	*225 (38.3)*	52 (28.3)	228 (33.3)	
BMI (kg/m^2^)					*0.030*
< 25	681 (49.9)	328 (*56.6*)	94 (50.5)	355 (53.4)	
25–29	475 (34.8)	187 (32.2)	62 (33.3)	236 (35.5)	
≥ 30	208 (*15.3*)	65 (11.2)	30 (*16.1*)	74 (11.1)	
Physical activity (times/week)					*0.038*
< 2–3 times	983 (69.9)	379 (64.6)	138 (74.2)	467 (67.9)	
≥ 2–3 times	423 (30.1)	208 *(35.4*)	48 (25.8)	221 (32.1)	
Smoking habits					0.577
No smoker	684 (49.4)	305 (52.6)	81 (43.8)	333 (49.3)	
Ex smoker	411 (29.7)	158 (27.2)	60 (32.4)	200 (29.6)	
Current smoker	290 (20.9)	117 (20.2)	44 (23.8)	142 (21.0)	
Alcohol intake (No/Yes)					0.158
No drinker	875 (62.3)	349 (59.4)	121 (64.7)	399 (58.1)	
Drinker	529 (37.7)	239 (40.6)	66 (35.3)	288 (41.9)	
Atopy					< 0.001*
No	931 (*72.7*)	–	188 (100.0)	268 (41.9)	
Yes	349 (27.3)	592 (100.0)	–	372 (*58.1*)	
Nasal polyps (ever)					< *0.001*
No	1396 (98.8)	564 (95.4)	185 (98.4)	632 (91.7)	
Yes	17 (1.2)	*27 (4.6)*	3 (1.6)	*57 (8.3)*	
Asthma					< *0.001*
No	1807 (85.25)	225 (40.47)	109 (72.67)	328 (52.82)	
Yes	107 (8.39)	*238 (42.81)*	*29 (19.33)*	*213 (34.30)*	
Past	81 (6.35)	*93 (16.73)*	12 (8.00)	*80 (12.88)*	
Chronic chough and phlegm					< *0.001*
No	1.338 (95.1)	524 (88.81)	154 (83.24)	588 (85.59)	
Yes	69 (4.9)	*66 (11.19)*	*31 (16.76)*	*99 (14.41)*	
Doctor-diagnosed chronic bronchitis					0.562
No	1236 (97.78)	521 (97.38)	156 (96.89)	587 (96.71)	
Yes	28 (2.22)	14 (2.62)	5 (3.11)	20 (3.29)	
Steroids (ever)					< *0.001*
No	1302 (98.6)	422 (80.8)	160 (92.5)	491 (79.1)	
Yes	18 (1.4)	*100 (19.2)*	*13 (7.5)*	*130 (20.9)*	
Vasoconstrictors (ever)					< *0.001*
No	1274 (96.4)	394 (75.3)	142 (81.6)	486 (77.8)	
Yes	48 (3.6)	*129 (24.7)*	*32 (18.4)*	*139 (22.2)*	
Antihistamines (ever)					< *0.001*
No	1320 (99.7)	362 (69)	162 (93.1)	495 (78.8)	
Yes	4 (0.3)	*163 (31.1)*	*12 (6.9)*	*133 (21.2)*	

Significance of differences was computed by Fisher’s exact test or Chi-square test

^*^Computed only on controls and people with sinusitis

### Description of controls and cases of gastroesophageal disorders, as a function of main risk factors

People with gastritis and/or gastroesophageal reflux were older and had attained a lower education level than people without these disorders (Table 2). Women more frequently reported gastritis and gastroesophageal reflux than men. Gastritis and gastroesophageal reflux were more common, respectively, in current smokers and overweight people.

**Table 2:** Number (percentage) of controls, cases of gastritis and gastroesophageal reflux, isolated or combined, as a function of main socio-demographic, lifestyle and clinical characteristics

	No gastritis/reflux (n = 2128)			Gastritis only (n = 264)			Reflux only (n = 310)	Both Gastritis and Reflux (n = 278)		*p *value
Sex										*0.034*
Male	1101 (51.7)			131 (49.6)			159 (51.3)	118 (42.5)		
Female	1027 (48.3)			133 (50.4)			151 (48.7)	*160 (57.6)*		
Age (years)										< *0.001*
20–34	*282 (13.3)*			11 (4.2)			19 (6.1)	15 (5.4)		
35–44	601 (28.2)			61 (23.1)			78 (25.2)	70 (25.2)		
45–54	648 (30.5)			68 (25.8)			87 (28.1)	81 (29.1)		
55–64	323 (15.2)			46 (17.4)			*61 (19.7*)	*63 (22.7)*		
> 65	274 (12.9)			*78 (29.6)*			*65 (21.0)*	*49 (17.6)*		
Time at stopping education (years)										< *0.001*
< 18	578 (27.4)			*111 (42.4)*			93 (30.4)	*103 (37.5)*		
18–21	806 (38.2)			73 (27.9)			111 (36.3)	99 (36.0)		
≥ 22	727 (34.4)			78 (29.8)			102 (33.3)	73 (26.5)		
BMI										*0.021*
< 25	1104 (53.8)			137 (53.5)			130 (43.6)	126 (46.8)		
25–29	679 (33.1)			83 (32.4)			125 (*42.0*)	102 (37.9)		
≥ 30	271 (13.2)			36 (14.1)			43 (14.4)	41 (*15.2*)		
Physical activity (hours/week)										0.112
< 2/3	1428 (67.7)			194 (74.0)			217 (70.5)	198 (71.5)		
≥ 2/3	683 (32.4)			68 (26.0)			91 (29.5)	79 (28.5)		
Smoking habits										*0.006*
No smoker	1059 (50.9)			103 (40.4)			154 (50.2)	128 (47.4)		
Ex smoker	588 (28.2)			92 (36.1)			105 (34.2)	79 (29.3)		
Current smoker	435 (20.9)			*60 (23.5)*			48 (15.6)	*63 (23.3)*		
Alcohol intake (no/yes)										0.793
No drinker	1284 (60.9)			165 (63.2)			182 (59.1)	168 (60.4)		
Drinker	826 (39.1)			96 (36.8)			126 (40.9)	110 (39.6)		
Atopy										0.485
No	1009 (51.1)			123 (53.0)			131 (49.6)	143 (55.6)		
Yes	964 (48.9)			109 (47.0)			133 (50.4)	114 (44.4)		
Nasal polyps										0.238
No	2049 (96.6)			253 (96.2)			291 (94.5)	271 (97.5)		
Yes	73 (3.4)			10 (3.8)			17 (5.5)	7 (2.5)		

Significance of differences was evaluated by Fisher’s exact test or Chi-square test

Atopy and nasal polyps, the level of physical activity and alcohol intake did not significantly change as a function of gastritis/gastroesophageal reflux.

### Description of controls and cases of AR/NAR/Sinusitis, as a function of gastroesophageal disorders

Gastritis/gastroesophageal reflux were strongly associated with nasal disorders. The prevalence of gastritis/reflux was 5.9% in controls, it slightly increased to 7.5% in cases of allergic rhinitis and further to 18.1% and 15.8% in cases of NAR and sinusitis, respectively (Table 3).

**Table 3:** Prevalence of gastroesophageal disorders (gastritis/GERD) in controls, rhinitis, allergic rhinitis and sinusitis subjects

	Controls (n = 1414) n(%)	AR (n = 590) n(%)	NAR (n = 188) n(%)	Sinusitis (n = 691) n(%)	*p* value
Gastritis/reflux					< *0.001*
No gastritis/GERD	1091 (77.2)	438 (74.2)	119 (63.3)	415 (60.1)	
Gastritis/GERD	323 (22.8)	152 (25.8)	69 (36.7)	276 (39.9)	
Gastritis only	108 (7.6)	43 (7.3)	21 (11.2)	81 (11.7)	
GERD Only	132 (9.3)	65 (11)	14 (7.5)	86 (12.5)	
GERD and Gastritis	83 (5.9)	44 (7.5)	34 (18.1)	109 (15.8)	

*p* values were computed by Pearson's Chi-square test

### Multivariable analyses

**Fig. 1:**
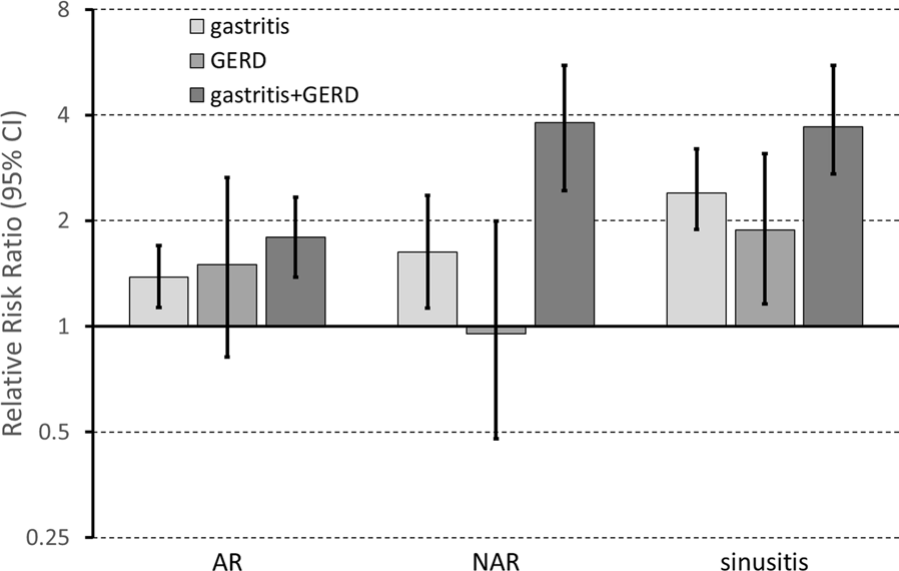
Association between nasal disorders and gastritis/gastroesophageal reflux. Relative Risk Ratios (RRR) were estimated by a multinomial regression model, adjusting for sex, age, education level, BMI, smoking habits, alcohol intake. Columns are RRRs, bars are 95% confidence inervals

The combination of gastritis and GERD was associated with a four-fold increase in the risk of NAR (RRR = 3.80, 95% CI 2.56–5.62) and sinusitis (RRR = 3.70, 2.62–5.23) with respect to people without these disorders, and with a much smaller increase in the risk of AR (RRR = 1.79, 1.37–2.35). The risk of nasal disorders was significanlty increased, although to a smaller extent, in subjects reporting gastritis alone, while GERD was significantly associated with sinusitis but not with either NAR or AR (Fig. 1).

A regards the other factors, the risk of NAR was higher in women than men (RRR = 1.28, 1.19–1.37). Moreover, the risk of AR and sinusitis decreased with advancing age (RRR per 10-year increase = 0.70, 0.63–0.79 and 0.79, 0.66–0.95, respectively). The risk of NAR was higher in current smokers than never smokers (RRR = 1.48, 1.16–1.89), and in people with medium than low education (RRR = 1.12, 1.00–1.26). With respect to normoweight, obesity was associated with a lower risk of AR (RRR = 0.75, 0.58–0.97) and sinusitis (RRR = 0.79, 0.65–0.95). The risk of sinusitis was slightly higher in past smokers than never smokers (RRR = 1.18, 1.01–1.38) and in alcohol drinkers than non-drinkers (RRR = 1.23, 1.02–1.47).

## Discussion

The main findings of the present study are:

1. Gastritis and GERD were strongly associated with non-allergic nasal diseases (NAR and sinusitis) and, to a much smaller extent, to allergic rhinitis, both in univariable and multivariable analysis.
2. The association was rather strong for the combination of gastritis and GERD, and rather weak for gastritis alone, and nearly absent for GERD alone. Subjects self-reporting both gastrointestinal diseases had probably a more severe condition than the other subjects.
3. Nasal polyps, while nearly absent in controls and cases of NAR, were found in 5% of cases of AR and 8% of cases of sinusitis. Their prevalence was not significantly affected by gastritis/GERD.
4. The risk of AR and sinusitis decreased with advancing age and in obese people. As regards lifestyle factors, NAR was associated with current smoking, and sinusitis with alcohol consumption.

The present multicase-control study showed that upper gastrointestinal disorders were strongly associated with non-allergic nasal disorders: the prevalence of gastritis and/or GERD was 22.8% in controls, only slightly increased in cases of AR (25.8%), and peaked up in cases of non-allergic nasal disorders, such as NAR (36.7%) and sinusitis (39.9%). These findings were confirmed in multivariable analysis, where the combination of gastritis and GERD was associated with a nearly four-fold increase in the risk of NAR and sinusitis, while the risk of AR was less than doubled.

These findings are in agreement with the current literature. As already mentioned, the association between chronic rhinosinusitis and GERD has been acknowledged by the International Consensus Statement on Allergy and Rhinology: Rhinosinusitis [20], although with moderate evidence. On the other hand, the association between AR and GERD is not even mentioned by the recent International Consensus Statement on Allergic Rhinitis [21]. A recent study supported GERD involvement in the development of NAR, since patients with NAR displayed a high level of pepsin in saliva samples, especially in the postprandial period, compared to healthy controls [4].

Nasal polyps were not significantly related to gastritis/GERD in the present study. According to Lin et al. [17] the association between GERD and chronic rhinosinusitis was stronger in subjects without than with nasal polyps.

An interesting approach to the relation between GERD and nasal disorders consists in verifying whether treatment of GERD could improve nasal symptoms especially in chronic rhinosinusitis refractory to clinical or surgical treatment. However, a recent review assessed the effect of treatment with proton pump inhibitors (PPIs) on chronic rhinosinusitis symptoms in four longitudinal studies, and found conflicting results [25].

### Pathophysiological mechanisms

Several mechanisms have been proposed to explain the relation between acid reflux and chronic rhinosinusitis. Subjects with chronic rhinosinusitis have been shown to have more proximal gastroesophageal reflux than healthy controls [26].

First of all, gastric acid exposure may exacerbate inflammation within the mucosa of the upper airways and sinuses and impair mucociliary motility, causing obstruction of sinus ostia and favouring recurrent infections [27–29].

A second mechanism could be vagally-mediated neuroinflammatory changes [11]. Autonomic dysfunction can lead to reflex sinonasal swelling and inflammation, leading to blockage of the ostia. Wong et al. [30] gave experimental support to this hypothesis, showing that infusion of saline with hydrochloric acid in the lower esophagus increased nasal mucus production and nasal symptom score.

Also a role of *Helicobacter pylori* (*H. pylori*) has been proposed, as the microorganism has been detected not only in the stomach but also in oral and nasal mucosa [31]. In particular, *H. pylori* has been found in nasal polyps but not in control tissue [32], and in patients who have both GERD and chronic rhinosinusitis [33]. Moreover, *H. Pylori* causes not only gastritis but also systemic inflammation, which can involve also the nasal mucosa.

### Strengths and limitations

The present study has several strengths. It involved seven centres scattered from Northern to Southern Italy, 1,471 cases of nasal disorders and 1,416 controls, and information was collected by standardized methods (questionnaire and skin prick test).

However, some limitations should be acknowledged. First of all, the cross-sectional design did not allow to properly infer the cause-effect relationship between GERD/gastritis and NAR. Indeed, to prove such a cause effect relation, longitudinal studies with an adequate number of patients are needed. Second, while information on nasal disorders was based on questionnaire and objective measurement (skin prick test), information on gastritis and GERD was exclusively derived by questionnaire. Gastritis alone apparently had larger effects on AR and NAR than GERD alone. It should be reminded that the question on gastritis was probably more reliable as it involved not only medical diagnosis but also objective assessment (gastroscopy), while the question on GERD put together different diseases (GERD, hiatal hernia, or esophagitis) and did not refer to instrumentally confirmed diagnosis. Moreover, subjects who reported both gastritis and GERD probably had a more clear-cut gastrointestinal disease than those reporting only one disease. In turn, improvement in exposure definition allowed to better assess association with nasal disorders.

## Conclusions

According to the present study, gastritis and GERD were strongly associated with nasal disorders, in particular non-allergic ones (NAR and sinusitis). On the other hand AR, which was defined by symptoms and positive skin prick test, has an IgE-mediated pathogenesis and is only mildly affected by irritant substances, such as acid reflux.

Nevertheless, to prove a causal effect relationship, prospective studies with a significant number of patients are needed. In particular randomized controlled trials should verify whether reflux treatment also improve concomitant nasal disorders.

## Data Availability

The datasets used and/or analysed during the current study are available from the corresponding author on reasonable request.

## Abbreviations

GERD: Gastroesophageal reflux disease
NAR: Non-allergic rhinitis
AR: Allergic rhinitis
GEIRD: Gene-Environment Interactions in Respiratory Diseases
ECRHS: European Community Respiratory Health Survey
